# Cerebral Pulsatility Index and In-Hospital Mortality in Chinese Patients with Traumatic Brain Injury: A Retrospective Cohort Study

**DOI:** 10.3390/jcm11061559

**Published:** 2022-03-12

**Authors:** Tao Mei, Quan Zhou, Lie Chen, Zheyong Jia, Wei Xiao, Lixin Xu

**Affiliations:** 1 Department of Neurosurgical Care Unit, The First People’s Hospital of Changde City, Changde 415003, China; yilan1008@sina.com (T.M.); chenlie20062007@126.com (L.C.); 19173605610m@sina.cn (Z.J.); xiaoweihn@163.com (W.X.); 2 Ministry of Science and Education, The First People’s Hospital of Changde City, Changde 415003, China; zhouquan402@163.com

**Keywords:** pulsatility index (PI), in-hospital mortality, transcranial Doppler (TCD)

## Abstract

There are limited studies on the relationship between the vascular transcranial Doppler (TCD) pulsatility index (PI) and in-hospital mortality in patients with traumatic brain injury (TBI). To address this issue, we conducted this study to explore whether, in newly diagnosed Chinese TBI patients, the PI is an independent predictor of the in-hospital mortality rate after adjusting for other covariates. This study is a retrospective cohort study. From 24 March 2019 to 24 January 2020, we recruited 144 Chinese patients with newly diagnosed TBI from a Chinese hospital. The independent variable was the PI, and the dependent variable was in-hospital mortality in TBI patients. The relationship between the PI and in-hospital mortality in TBI patients was nonlinear and had an inflection point of 1.11. In the multivariate analysis, after adjusting for potential confounders, the effect sizes and confidence intervals per additional 0.1 units on the left and right sides of the inflection point were 4.09 (1.30–12.83) and 1.42 (0.93–2.17). The relationship between the PI and in-hospital mortality was nonlinear. The PI was positively related with in-hospital mortality when the PI was less than 1.11.

## 1. Introduction

TBI can be classified into three grades: mild, moderate, and severe. According to data from the World Health Organization, approximately 70–90% of TBI patients are of mild grade [1,2]. The presence of several major post-traumatic cerebral hemodynamic disturbances results in a poor prognosis of secondary craniocerebral injury. To date, the accuracy of the traditional scaling methods for TBI severity is occasionally affected by factors such as sedation, intubation, and limb movement disorder of the patient, resulting in difficulties in clinical evaluation. As an objective, non-invasive, and reproducible method, TCD can be used as a bedside evaluation for cerebral hemodynamic changes in the intracranial arterial system. TCD has been widely used in neurosurgery patients, especially those with severe TBI [3,4]. The PI is a TCD parameter closely related to the severity of cerebral hemodynamic disorder, and an abnormal PI itself can be independently related to the patient’s mortality [5,6]. Previous studies have shown that PI has significant correlations with the prognostic factors in TBI patients including intracranial pressure (ICP) and cerebral perfusion pressure (CPP) (PI is positively proportional to the ICP and inversely proportional to the average CPP) [7,8]. Therefore, some studies have pointed out the importance of cerebral monitoring, such as TCD, to avoid secondary brain injury [9]. The present study included 144 TBI patients in a neurosurgical care unit (NCU) in China. With data collection and analysis, the relationship between TCD PI and in-hospital mortality of TBI patients was retrospectively studied.

## 2. Materials and Methods

### 2.1. Research Design

We conducted a retrospective study to address the relationship between the PI and in-hospital mortality of TBI patients. The independent variable was the PI, and the dependent variable was the in-hospital mortality of TBI patients.

### 2.2. Study Population

Data on Chinese TBI patients were continuously collected in a non-selective manner from the NCU of the First People’s Hospital of Changde City, Hunan. Data that could be used to identify patients were excluded to protect patients’ privacy. The data were obtained from the electronic medical record system of the hospital. This study did not require informed consent from the participants because it was retrospective in nature. This study was approved by the hospital institutional review board to initially collect data from a total of 195 participants. The period of data collection was from 24 March 2019 to 24 January 2020.

All patients were transferred from the Emergency Department to the NCU in the same hospital. The inclusion criteria for patients were as follows: computerized tomography-verified TBI (subdural hematoma, parenchymal hematoma, cerebral contusion and laceration, traumatic subarachnoid hemorrhage, and epidural hematoma); age > 15 years; hospitalization time longer than 24 h. The exclusion criteria were absence of TCD on the middle cerebral artery (MCA) due to the fact of poor transtemporal windows; surgical bandage or soft tissue hematoma; irregular left ventricular ejection volume (such as atrial fibrillation and atrial flutter as well as frequent ventricular premature beats); and heart rate greater than 140 beats per minute.

### 2.3. Trauma Type and Treatment

The median age of the 144 patients was 45 years (range: 15–75 years). The main cause of trauma was car accidents (101 cases, 70%), followed by falls (35 cases, 24%), and falls from a height (8 cases, 6%). Twenty-five (17%) of the 144 patients had multiple injuries. There were 20 cases of bone fractures, 8 cases of chest injuries, and 3 cases of spinal injuries. Five patients (3%) suffered multiple life-threatening injuries. Ninety-five patients (66%) underwent early surgery due to the presence of mass lesions, such as acute intracranial hematomas, and 49 patients (34%) were treated non-surgically. Among the 95 surgical patients, 20 had epidural hematoma, 90 had acute subdural hematoma, 50 had intracerebral hematoma, and 90 had traumatic subarachnoid hemorrhage. Fifty-five out of the ninety-five surgical patients received DC. In terms of the extent of injury, 15 of the 49 conservatively treated cases were diffuse axonal injury. The brain trauma in 122 patients were located at the supratentorial area, and 22 patients had combined injuries in both the supratentorial and infratentorial areas. All patients received standardized treatment in the NCU: ICP ≤ 20 mmHg, CPP ≥ 60 mmHg, systolic blood pressure > 100 mmHg, central venous pressure between 0 and 5 mmHg, P0 > 12 KPa, blood glucose between 5 and 10 mmoL/L, normal electrolyte range, and a body temperature <38 °C.

### 2.4. Measurement of the PI Value

TCD uses a low-frequency (2 MHz) transducer on the scalp surface to measure the M1–M2 segment of the bilateral MCA through the transtemporal window. For each side measurement, the TCD inspection involved continuous monitoring for 30 min, and the average measured value of echo depth was selected. The data obtained consisted of the mean blood flow velocity (MV), peak blood flow velocity (PV), and end-diastolic blood flow velocity (EDV). The PI was calculated as (PV − EDV)/MV, According to previous study normal TCD pattern was defined as PI less than 1.25 [10] or 1.4 [11], but data about the thresholds used to define abnormal TCD are rare, and we mainly had to rely on our own clinical experience, and a PI ≤ 1 was considered normal [12,13]. We recorded the baseline PI as a continuous variable. A detailed description is as follows: (1) If the vital signs of the patient were stable, TCD measurement was performed within 24 h after admission to the hospital; otherwise, patients received TCD as soon as possible after hemodynamic stability was achieved. (2) All TCD measurements were conducted by technicians from the Ultrasound Department in our hospital using the DWL Multi-Dop 2 (Germany) instrument. Blood biochemical measurements were conducted in the laboratory at our hospital within 24 h after admission. (3) To maintain reproducibility of the records, the patient was stable, i.e., no agitation or pain, and no cardiopulmonary distress.

### 2.5. Covariates

The covariates used in this study can be categorized as follows: (1) demographic data; (2) variables affecting PI and in-hospital mortality according to previous literature; and (3) variables determined based on our clinical experience.

### 2.6. Statistical Analysis

The continuous variables were presented in two ways. Those with normal distribution were presented as the mean ± standard deviation, and others with skewed distribution as medians and interquartile ranges (Q1–Q3). Categorical variables were described using frequencies or percentages. The χ^2^ test (categorical variables), the one-way ANOVA (normal distributed variables), or the Kruskal–Wallis H test (skewed distributed variables) were used to test the differences between PI groups.

The entire data analysis process can be divided into two steps. Step 1: The use of univariate and multivariate logistic regression methods. We established three models: Model 1 did not include covariate adjustment; Model 2 only included adjustments for demographic data; and Model 3 included adjustments for the covariates shown in Table 1. Variables based on epidemiological and biological backgrounds were combined into potential confounding factors, and those confounding factors adjusted for changes in the estimated effect of >10% were used to generate the adjusted model. Step 2: Exploration of the nonlinear relationship between PI and in-hospital mortality and performance of a smooth curve fit. If nonlinearity was detected, we first used a recursive algorithm to calculate the inflection point and then constructed a two-stage linear regression model on both sides of the inflection point. We determined the best-fit model based on the *p*-value of the log-likelihood ratio test with the aim of verifying the results with PI as a continuous variable and to observe the possibility of nonlinearity. All analyses were performed with the R statistical package (http://www.R-project.org (accessed on 1 October 2020), The R Foundation) and Empower Stats (http://www.empowerstats.com (accessed on 1 October 2020), X & Y Solutions, Inc., Boston, MA, USA). *p*-values less than 0.05 (two-sided) were considered statistically significant.

## 3. Results

### 3.1. Participant Selection

**Figure 1:**
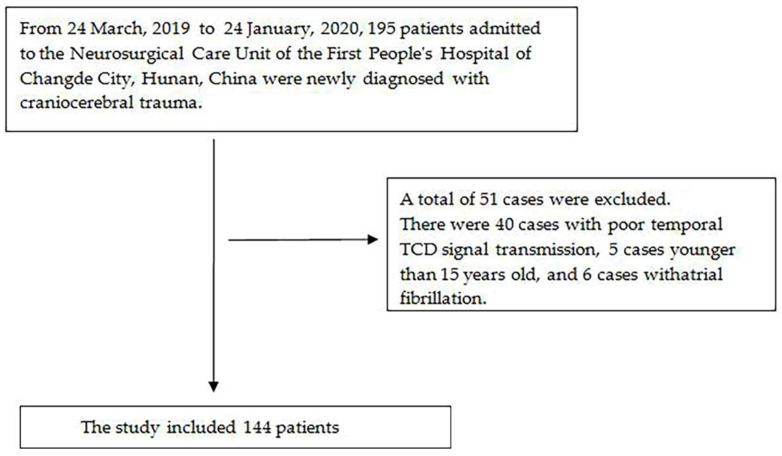
Flow chart.

Based on our strict screening criteria, a total of 144 patients were selected for the final data analysis. A total of 51 cases were excluded. There were 40 cases with poor temporal TCD signal transmission, five cases younger than 15 years old, and six cases with atrial fibrillation (see Figure 1 for a flow chart).

### 3.2. Baseline Characteristics of the Selected Participants

Table 1 shows the baseline characteristics of the participants in each group. The mean age of the 144 selected TBI patients was 55.33 ± 16.26 years, consisting of 109 males (75.7%) and 34 females (24.3%). There was no statistical difference between the tertiles of PI in terms of age, trauma time before admission, hemoglobin count, Na^+^ concentration, gender, diabetes, hypertension, traumatic coagulopathy, sedation, and analgesia (all *p* > 0.05). Significant differences were detected in blood transfusion volume, PLR, the GCS on admission, shock, use of ventilator, PTCI, DC, and in-hospital mortality (all *p* < 0.05).

### 3.3. Results of the Univariate Logistic Analysis

The results of the univariate analysis are shown in Table 2. Univariate linear regression did not indicate a significant association between in-hospital mortality and age, gender, diabetes, hypertension, trauma time before admission, hemoglobin count, Na^+^ concentration, traumatic coagulopathy, sedation, and analgesia. In contrast, in-hospital mortality was associated with unilateral and bilateral PLR loss (OR 4.25; 95% CI 1.32–13.64) (OR 16.18; 95% CI 5.60–46.75); GCS (9–12 points) and GCS (3–8 points) (OR 0.20; 95% CI 0.06–0.62 and OR 0.18; 95% CI 0.05, 0.65); shock (OR 5.83; 95% CI 2.02–16.83); use of ventilator (OR 13.47; 95% CI 3.08–58.96); PTCI (OR 6.87; 95% CI 2.28–20.68); DC (OR 9.76; 95% CI 3.98–23.98); PI (β 1.85; 95% CI 1.48–2.31); and blood transfusion volume (β 1.00; 95% CI 1.00–1.00).

### 3.4. Results of theMultivariate Logistic Regression Models

In this study, we constructed three models to analyze the independent effects of each variable on in-hospital mortality (univariate and multiple linear regression). Table 3 lists the effect size (OR) and 95% confidence interval. In the unadjusted model (Model 1), the effect size can be interpreted as the correlation between the PI increase and in-hospital mortality. For example, in an unadjusted model, an effect size of 1.92 means that every 0.1 increase in the PI’s value is associated with a 0.92-fold increase in hospital mortality (OR 1.92; 95% CI 1.54–2.40). In the minimally adjusted model (Model 2), when the PI value increases by 0.1, the in-hospital mortality increases by 0.93 times (OR 1.93; 95% CI 1.54–2.42). In the fully adjusted model (Model 3) (adjusted for all covariates shown in Table 3), for every 0.1 increase in PI, the increase in in-hospital mortality is 0.88 times (OR 1.88; 95% CI 1.35–2.60).

### 3.5. The Nonlinear Relationship between PI and in-Hospital Mortality

**Figure 2:**
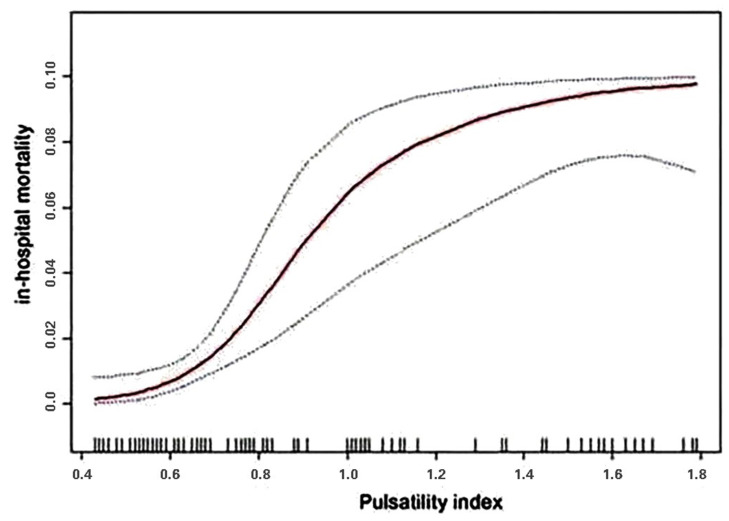
Association between PI and in-hospital mortality. The solid red line represents the smooth curve fit between the variables. Black dots indicate the 95% confidence bands of the fit. The model was adjusted for age, gender, diabetes, hypertension, PLR, GCS on admission, blood transfusion volume, PTCI, DC, use of ventilator, shock, hemoglobin count, Na^+^ concentration, traumatic coagulopathy, sedation, and analgesia.

We analyzed the relationship between PI and in-hospital mortality in this study. The results of the smooth curve fitting and the generalized additive model (Figure 2) showed that the relationship between PI and in-hospital mortality was nonlinear. There was a saturation effect between PI and in-hospital mortality in two-piecewise linear regression analysis. After adjusting for age, gender, diabetes, hypertension, PLR, GCS on admission, blood transfusion volume, PTCI, DC, use of ventilator, shock, trauma time before admission, hemoglobin count, Na^+^ concentration, traumatic coagulopathy, sedation, and analgesia, the risk of in-hospital mortality only increased with an increasing PI value below the turning point (PI < 1.11) (Table 4; OR 4.09; 95% CI 1.30–12.83; *p* < 0.05).

## 4. Discussion

A large multicenter cohort study demonstrated acceptable sensitivity and specificity of TCD PI thresholds 1.25 for detecting early neurologic deterioration for mild to moderate TBI [14]. This study elucidates that the PI after admission was positively correlated with the in-hospital mortality of TBI patients. In addition, there was a threshold effect between PI and in-hospital mortality, and the inflection point was PI = 1.11, Basically consistent with the above PI threshold of 1.25. Independent risk factors for in-hospital mortality of patients included GCS on admission, PLR, shock, use of ventilator, DC, PTCI, blood transfusion volume, and PI. Our results are consistent with previous reports [15], except that traumatic coagulopathy related to in-hospital mortality did not present in our study. This was considered a result of the Emergency Department’s rapid correction of coagulation parameters early in the diagnosis and treatment [16].

The PI provides information on downstream cerebrovascular resistance and describes quantitative changes in the TCD waveform morphology. As a non-invasive evaluation, PI has been widely used in TBI patients for indirect estimation of ICP and CPP in clinical practice. The reliability of related studies [17] ensures the theoretical authenticity and reliability of this study.

Cerebral blood flow after TBI changes from hyper-acute hypoperfusion (day 0), to acute hyperemia (days 1–3), to late cerebral vasospasm (days 4–15), accompanied by intracranial pressure increases [18,19]. Intracranial hemodynamic changes lead to corresponding changes in PI. Higher PI values in the hospital for severe traumatic brain injury patients are related to brain hypoperfusion and higher in-hospital mortality [6]. The thresholds of 1.25 for PI accurately predicted neurologic worsening with 90% sensitivity and 91% specificity for mild to moderate TBI [10]. In children, TCD may be a useful tool to assess autoregulation, intracranial pressure, and vasospasm after TBI in the PICU. Further research is needed to establish the gold standard and validate findings in children [20]. By calculating linear regression equations, Splavski, 2006, demonstrated that each additional unit of PI value will be reduced by 2.613 units in the Glasgow Outcome Score (GOS) 6 months later [21]. PI and GOS showed similar trends at 3 and 12 months after TBI in our cohort study. However, the specific correlation has not been studied in depth, which is the direction of our deeper work. Bellner et al., 2004, found that there is a highly significant correlation between ICP and PI, regardless of specific intracranial lesions [22]. In a single-center study in China following decompressive craniectomy for TBI, there was no difference in mortality among patients in the ICP combined with TCD surveillance group, but more patients had favorable outcomes [11]. Our cohort study also observed an improvement in clinical symptoms after decompressive craniectomy, which was associated with a lower PI. In the systematic review conducted by Ziegler et al., 2017, PI shows significant predictive power on patient outcomes [6]. Similar to the above studies, our study also suggested that PI elevation was positively correlated with poor clinical outcomes. Christou I et al., 2001, found that elevated PI (PI > 1.56) in patients with severe TBI predicted adverse outcomes, and ICP and PI were further correlated [23]. In our study, the nonlinear relationship between PI value and in-hospital mortality was determined, and curve fitting was performed to calculate the inflection point. It was found that despite the adjustment of many confounding factors, there was still a correlation. When PI was below 1.11, the mortality rate increased by 3.09 times for each 0.1 unit increase in the PI, and when PI was above 1.11, the mortality rate increased by 0.42 times for each 0.1 unit increase in the PI. Compared with Christou I’s study, where PI > 1.56 indicated poor clinical prognosis, the prognosis threshold was not found in the segmented regression model used in our study. This is possibly due to the lower mean PI, in that most cases in this study were mild to moderate TBI (78%). Despite the above findings, only a few prospective cohort studies and randomized controlled trials have been reported so far. The diagnostic and prognostic roles of TCD in TBI have not yet been fully established, resulting in limited data [24]. Therefore, it is necessary to expand the scope of TBI research. Interestingly, a 2021 study found that [25] the “Dissimilarity/Difference Index” (DI) between transcranial Doppler flow velocity (FV) and arterial blood pressure (ABP) waveforms appears to be more robust than conventional pulsatility indices. Distinguish between ICP plateau waves and baseline episodes. This provides an idea for a new field to discover the predictive value of TBI prognosis.

The clinical significance of this study includes the following: (1) as far as we know, this is the first study to indicate a correlation between PI and in-hospital mortality of Chinese TBI patients and a threshold saturation effect; (2) according to this study, the stage when PI ≤ 1.11 had a great influence on in-hospital mortality, so it is suggested that the diagnosis and treatment of acute cerebrovascular dysfunction should be paid attention to in TBI treatment to improve the clinical prognosis; and (3) this study will be helpful for establishing prediction models for TBI patients.

The advantages of this study are as follows: (1) in this study, the piecewise relationship between risk factors and the dependent variable was detected using smooth curve fitting, and the nonlinearity problem was solved by the piecewise linear regression model; and (2) strict statistical adjustments were used in this study to minimize the residual confounding factors to which observational studies are susceptible.

The limitations of this study are as follows: (1) This study only included Chinese TBI patients, and its general applicability is not strong. The number of cases, especially cases with severe TBI, was not large, which could lead to statistical deviation. (2) There was a reliance on a clinical TCD operator and limited spatial resolution; the poor transtemporal windows in a portion of cases [26,27,28] may have affected clinical operations.

## 5. Conclusions

In conclusion, this study showed that the PI of Chinese TBI patients may have a positive correlation with in-hospital mortality, and the correlation may have a segmented threshold effect.

## Figures and Tables

**Table 1:** Baseline characteristics of the participants. (*n* = 144).

Characteristics	PI Tertiles			*p*-Value
	T1 (0.56–0.81)	T2 (0.82–1.01)	T3 (1.02–2.11)	
Number of participants	47	48	49	
Age (years, mean ± SD)	52.34 ± 15.77	54.60 ± 15.95	59.71 ± 14.38	0.058
Trauma time before admission (hours, median, Q1–Q3)	15.53 ± 42.46	10.82 ± 17.99	12.74 ± 26.43	0.753
Hemoglobin count (g/L, mean ± SD)	118.13 ± 20.17	116.08 ± 20.94	113.63 ± 22.25	0.582
Na^+^ concentration (mmol/L, mean ± SD)	139.48 ± 3.53	139.60 ± 4.16	140.24 ± 4.27	0.604
Blood transfusion volume (mL, median, Q1–Q3)	158.72 ± 306.33	361.70 ± 607.31	954.08 ± 885.70	<0.001
Gender				0.478
Male	33 (70.21%)	38 (79.17%)	39 (79.59%)	
Female	14 (29.79%)	10 (20.83%)	10 (20.41%)	
Diabetes				0.732
No	45 (95.74%)	45 (93.75%)	45 (91.84%)	
Yes	2 (4.26%)	3 (6.25%)	4 (8.16%)	
Hypertension				0.182
No	44 (93.62%)	39 (81.25%)	41 (83.67%)	
Yes	3 (6.38%)	9 (18.75%)	8 (16.33%)	
PLR				<0.001
Bilateral PLR exhibition	42(89.36%)	41 (85.42%)	22 (44.90%)	
Unilateral PLR loss	5 (10.64%)	4 (8.33%)	7 (14.29%)	
Bilateral PLR loss	0 (0.00%)	3 (6.25%)	20 (40.82%)	
GCS				<0.001
3–8	11 (23.40%)	26 (54.17%)	38 (77.55%)	
9–12	18 (38.30%)	14 (29.17%)	6 (12.24%)	
13–15	18 (38.30%)	8 (16.67%)	5 (10.20%)	
Shock				0.015
No	45 (95.74%)	44 (91.67%)	38 (77.55%)	
Yes	2 (4.26%)	4 (8.33%)	11 (22.45%)	
Traumatic coagulopathy				0.716
No	39 (82.98%)	40 (83.33%)	38 (77.55%)	
Yes	8 (17.02%)	8 (16.67%)	11 (22.45%)	
Sedation and analgesia				0.812
No	2 (4.26%)	1 (2.08%)	2 (4.08%)	
Yes	45 (95.74%)	47 (97.92%)	47 (95.92%)	
Use of ventilator				<0.001
No	25 (53.19%)	22 (45.83%)	4 (8.16%)	
Yes	22 (46.81%)	26 (54.17%)	45 (91.84%)	
PTCI				<0.001
No	47 (100.00%)	44 (91.67%)	37 (75.51%)	
Yes	0 (0.00%)	4 (8.33%)	12 (24.49%)	
DC				<0.001
No	37 (78.72%)	37 (77.08%)	15 (30.61%)	
Yes	10 (21.28%)	11 (22.92%)	34 (69.39%)	
In-hospital mortality				<0.001
No	47 (100.00%)	45 (93.75%)	17 (34.69%)	
Yes	0 (0.00%)	3 (6.25%)	32 (65.31%)	

GCS: Glasgow Coma Scale; PLR: pupillary light reflex; DC: decompressive craniectomy; PTCI: post-traumatic cerebral infarction; PI: pulsatility index. Values are the mean± standard deviation or *n* (%).

**Table 2:** Univariate analysis for in-hospital mortality.

Covariate	Statistics	In-Hospital Mortality	
Age, years	55.60 ± 15.58	1.03 (1.00, 1.06)	0.0759
Gender			
Male	110 (76.39%)	Reference	
Female	34 (23.61%)	1.16 (0.48, 2.80)	0.7364
Diabetes			
No	135 (93.75%)	Reference	
Yes	9 (6.25%)	2.68 (0.68, 10.61)	0.1592
Hypertension			
No	124 (86.11%)	Reference	
Yes	20 (13.89%)	1.04 (0.35, 3.11)	0.9378
PLR			
Bilateral PLR exhibition	105 (72.92%)	Reference	
Unilateral PLR loss	16 (11.11%)	4.25 (1.32, 13.64)	0.0151
Bilateral PLR loss	23 (15.97%)	16.18 (5.60, 46.75)	<0.0001
GCS			
13–15	75 (52.08%)	Reference	
9–12	38 (26.39%)	0.20 (0.06, 0.62)	0.0052
3–8	31 (21.53%)	0.18 (0.05, 0.65)	0.0086
Shock			
No	127 (88.19%)	Reference	
Yes	17 (11.81%)	5.83 (2.02, 16.83)	0.0011
Use of ventilator			
No	51 (35.42%)	Reference	
Yes	93 (64.58%)	13.47 (3.08, 58.96)	0.0006
PTCI			
No	128 (88.89%)	Reference	
Yes	16 (11.11%)	6.87 (2.28, 20.68)	0.0006
DC			
No	89 (61.81%)	Reference	
Yes	55 (38.19%)	9.76 (3.98, 23.98)	<0.0001
PI	1.03 ±0.45	1.85 (1.48, 2.31)	<0.0001
Blood transfusion volume, mL	497.97 ± 728.75	1.00 (1.00, 1.00)	<0.0001
Trauma time before admission, hours	13.01 ± 30.41	0.95 (0.90, 1.01)	0.0773
Hemoglobin count, g/L	115.92 ± 21.09	0.99 (0.97, 1.01)	0.2110
Na+ concentration, mmol/L	139.78 ± 3.99	0.94 (0.85, 1.03)	0.1872
Traumatic coagulopathy			
No	117 (81.25%)	Reference	
Yes	27 (18.75%)	1.40 (0.55, 3.56)	0.4756
Sedation and analgesia			
No	5 (3.47%)	Reference	
Yes	139 (96.53%)	1.30 (0.14, 11.99)	0.8198

GCS: Glasgow Coma Scale; PLR: pupillary light reflex; DC: decompressive craniectomy; PTCI: post-traumatic cerebral infarction; PI: pulsatility index. Values are the mean± standard deviation or *n* (%).

**Table 3:** Relationship between PI and in-hospital mortality in the different models.

Variable OR (95% CI) *p*-Value	Non-Adjusted OR (95% CI) *p*-Value	Adjust I OR (95% CI) *p*-Value	Adjust II OR (95% CI) *p*-Value
PI *	1.92 (1.54, 2.40) <0.0001	1.93 (1.54, 2.42) <0.0001	1.88 (1.35, 2.60) <0.001

* Per 0.1 units added. PI: pulsatility index; CI: confidence interval. Model I was adjusted for age and gender. Model II was adjusted for age, gender, diabetes, hypertension, PLR, GCS on admission, blood transfusion volume, PTCI, DC, use of ventilator, shock, trauma time before admission, hemoglobin count, Na^+^ concentration, traumatic coagulopathy, sedation, and analgesia.

**Table 4:** Univariate analysis for PI using different models.

Inflection Point of PI	Non-Adjusted OR (95% CI) *p*-Value	Adjust I OR (95% CI) *p*-Value	Adjust II OR (95% CI) *p*-Value
<1.11 *	4.19 (1.84, 9.56) 0.0006	4.30 (1.87, 9.88) 0.0006	4.09 (1.30, 12.83) 0.0158
≥1.11 *	1.41 (1.06, 1.86) 0.0163	1.40 (1.06, 1.86) 0.0172	1.42 (0.93, 2.17) 0.1064

* Per 0.1 units added. CI, confidence interval; OR, odds ratio. Model I was adjusted for age and gender. Model II was adjusted for age, gender, diabetes, hypertension, PLR, GCS on admission, blood transfusion volume, PTCI, DC, use of ventilator, shock, hemoglobin count, Na^+^ concentration, traumatic coagulopathy, sedation, and analgesia.

## Data Availability

The data used to support the findings of this study are available from the corresponding author upon request.
